# Ischaemic Stroke in Patients with Known Atrial Fibrillation: A Snapshot from a Large University Hospital Experience

**DOI:** 10.3390/jcm14176012

**Published:** 2025-08-25

**Authors:** Giulia Domna Scrima, Cristina Sarti, Giovanni Pracucci, Rita Nistri, Costanza Maria Rapillo, Benedetta Piccardi, Miroslava Stolcova, Francesca Ristalli, Alessio Mattesini, Carlo Nozzoli, Alessandro Morettini, Alberto Moggi Pignone, Patrizia Nencini, Carlo Di Mario, Rossella Marcucci, Francesco Meucci

**Affiliations:** 1 Department of Neurofarba, University of Florence, 50134 Florence, Italy; giuliadomna.scrima@unifi.it (G.D.S.);; 2 Stroke Unit, Careggi University Hospital (AOUC), 50139 Florence, Italy; benedetta.piccardi@unifi.it (B.P.); nencinip@aou-careggi.toscana.it (P.N.); 3 Department of Heart and Vessels, Careggi University Hospital (AOUC), 50134 Florence, Italy; nistrir@aou-careggi.toscana.it; 4 Neurology and Stroke Unit, Istituto di Ricovero e Cura a Carattere Scientifico, Humanitas Research Hospital, 20089 Rozzano, Italy; costanza.rapillo@humanitas.it; 5 Structural Interventional Cardiology, Careggi University Hospital (AOUC), 50134 Florence, Italy; stolcovam@aou-careggi.toscana.it (M.S.); ristallif@aou-careggi.toscana.it (F.R.); mattesinia@aouc-careggi.toscana.it (A.M.); carlo.dimario@unifi.it (C.D.M.); meuccif@aou-careggi.toscana.it (F.M.); 6 Internal Medicine 1, Careggi University Hospital (AOUC), 50134 Florence, Italy; nozzolic@aou-careggi.toscana.it; 7 Internal Medicine 2, Careggi University Hospital (AOUC), 50134 Florence, Italy; morettinia@aou-careggi.toscana.it; 8 Department of Experimental and Clinical Medicine, University of Florence, 50134 Florence, Italy; alberto.moggipignone@unifi.it; 9 Atherothrombotic Diseases, Careggi University Hospital (AOUC), 50134 Florence, Italy; rossella.marcucci@unifi.it

**Keywords:** atrial fibrillation, oral anticoagulant therapy, resistant stroke

## Abstract

**Objectives**: Atrial fibrillation (AF) is associated with high risk of ischaemic stroke (IS). Oral anticoagulant therapy (OAT) is the standard of care for stroke prevention, even though its management remains challenging in clinical practice. An emerging problem is embolic events occurring on adequately conducted OAT, the so-called resistant stroke (RS). We aimed to describe pre-stroke prevention therapy, management on hospital discharge, and therapy at follow-up in all patients with AF hospitalized for IS and in the RS subgroup. **Methods**: We conducted a retrospective monocentric study of patients with known AF hospitalized for an IS. A subgroup with RS was identified. We recorded information on prevention therapy at home, recommended therapy at discharge, and data on outcome and prevention therapy at follow-up. **Results**: We identified 226 patients, 61% females, median age 84.04 years. Preventive therapy at home was performed in 121 (53.5%) (119 OAT and 2 Left Atrial Appendage Occlusion). At hospital discharge OAT was prescribed to 78.2% of patients. RS was diagnosed in 33 patients whose management at discharge was: same OAT in 12, shift to another Direct Oral Anticoauglant (DOAC) in 5, from DOAC to Vitamin K Antagonist (VKA) and vice versa in 11, non-specified OAT in 4. At final, follow-up of 208 days (range 85–443) 23.3% (34/146) did not assume OAT. OAT was significantly associated with survival probability (*p* < 0.001). **Conclusions**: Our findings confirm a scarce adoption of guidelines for AF-related embolic events, even in the absence of absolute contraindication to OAT. RS remains an underexplored clinical entity with empirical management, highlighting the need for targeted research and tailored therapeutic strategies.

## 1. Introduction

Atrial fibrillation (AF) is one of the most common cardiac arrhythmias [1], and it is associated with a five-fold increased risk of ischaemic stroke (IS) [2]. Strokes attributable to AF are associated with high severity, a significant burden of disability, and an elevated risk of recurrence [3]. Oral anticoagulant therapy (OAT), either with a vitamin K antagonist (VKA) or a direct oral anticoagulant (DOAC), is the most effective strategy for preventing IS in patients with AF, as it reduces both the risk and the severity of embolic events [4].

The use of oral anticoagulants in clinical practice faces several well-recognized challenges:

(1) under-prescription, particularly among the elderly even without clear contraindication
(2) suboptimal management, i.e., difficulties in keeping INR in range for patients treated with VKA or wrong dosage/poor compliance for patients treated with DOAC
(3) a failure of OAT (i.e., patients facing ischaemic stroke due to AF despite an adequate conducted anticoagulant therapy), is a well-recognized phenomenon in the literature. This condition has been referred to as ‘resistant stroke’ (RS) [5], and in this study we will adopt this term to describe such cases. The mechanisms underlying RS remain poorly understood, and consequently, secondary prevention in these patients is largely empirical.

Our aims are:

1. To describe the primary and secondary prevention strategies undertaken in patients with known AF and acute ischaemic cerebral event.
2. To estimate the percentages of RS and its management.

## 2. Materials and Methods

### 2.1. Study Design and Population

We conducted a single centre retrospective study including all consecutive patients admitted for IS or transient ischaemic attack (TIA) with a known history of AF to the Stroke Unit and Internal Medicine Departments of Careggi University Hospital, Florence, Italy, between 1 July 2017 and 30 June 2019.

### 2.2. Data Collection

Data were obtained from medical records and included:

– demographic variables: age and sex
– cardiovascular risk factors: history of hypertension, diabetes, smoking habit, dyslipidaemia, history of heart failure
– previous cerebrovascular events: TIA, ischaemic or haemorrhagic stroke
– echocardiographic data: ejection fraction, left atrial enlargement, valve defects and relative grade, presence of prosthetic heart valves. Ejection fraction was considered normal when ≥50%; left atrial enlargement was dichotomised as yes or no and was defined as an increase of diameter or area or volume, valve defects were indicated as stenosis (mild, moderate or severe), insufficiency or combined
– cervical and intracranial vessels hemodynamic stenosis assessed by CT angiography or doppler ultrasonography
– site and size of ischaemic cerebral lesion on cerebral CT scan and/or cerebral MRI
– etiopathogenesis of ischaemic cerebrovascular events was classified according to TOAST criteria [6]. Embolic stroke, after exclusion of alternative etiologies, were classified as cardioembolic based on the known AF. When more than one potential embolic source was identified, the stroke etiology was classified as “undetermined”. Etiology classification was performed by expert vascular neurologists.
– CHA_2_DS_2_-VASc and HAS-BLED score for evaluating thromboembolic and haemorrhagic risk respectively [7]
– Pre-event disability assessed by modified Rankin Scale (mRS)
– Type of preventive therapy on admission, and at discharge:
  (1) OAT: VKA or DOAC
  (2) Left Atrial Appendage Occlusion (LAAO)
  (3) No preventive therapy

### 2.3. Definition of Adequate OAT

Data on adequate OAT were collected and defined according to the following criteria:

- For patients on VKA, therapy was considered adequate when the INR at hospital admission was ≥1.7, which corresponds to the internationally accepted cut-off for eligibility to systemic thrombolysis [8].
- For patients on DOAC, adequacy was defined when both the indication and dosage were in accordance with the current European guidelines [9] and when good adherence was either reported by the patient/caregiver or confirmed by plasmatic drug level measurement. When information about compliance was unavailable, patients were classified in an “uncertain” group.

### 2.4. Definition of RS

We defined RS as occurring when AF was the only possible cause of stroke in patients with adequate OAT; patients with mechanical valve and severe mitral stenosis were excluded.

### 2.5. Follow-Up

A telephone follow-up was carried out to register:

– Adoption of secondary prevention therapy for cardioembolic stroke recommended at hospital discharge
– Recurrence of ischaemic or haemorrhagic stroke
– Death and its cause.

This study was conducted in accordance with ethical guidelines for observational cohort studies.

## 3. Statistical Analysis

Categorical variables were reported as proportions, and differences between groups were assessed using Chi-square test. Continuous variables were expressed as median and interquartile range (IQR) and compared using Mann-Whitney U test, given their non-normal distribution. To identify factors independently associated with mortality at follow-up, variables with *p* < 0.10 in univariate analysis were entered into a multivariable logistic regression model. Survival according to OAT was estimated using the Kaplan Meier method, with differences assessed with the Log-Rank test. Cox proportional hazards regression was applied to adjust survival data for age and stroke severity (admission NIHSS); a sensitivity analysis was performed in the subset of patients with available pre-stroke disability (mRS dichotomized as 0–2 vs. 3–5) which was included as an additional covariate in the Cox models. A two-tailed *p* value < 0.05 was considered statistically significant.

## 4. Results

### 4.1. Population Characteristics

We enrolled 226 patients (218 stroke and 8 TIA), 61% of whom were females, the median age was 84.04 (IQR 77.9–88.) years. The distribution of cardiovascular risk factors is reported in Table 1. The median CHA_2_-DS_2_-VASc was 4.5 (IQR: 3–5), and all but one in patient had indication for OAT.

Stroke pathogenesis was classified as cardioembolic in 70.3%, small-artery occlusion in 2.2%, other determined causes in 0.9%, and undetermined in 26.5% (Table 1).

### 4.2. Preventive Therapy at Admission

On admission, 103 patients (45.6%) were on no preventive therapy, 119 (52.7%) were on OAT (72 VKA and 47 DOAC), 2 (0.9%) had undergone LAAO; data were missing for 2 patients (0.9%). Patients without preventive therapy were older (median age 85 years) and had higher CHA_2_DS_2_-VAsc (median = 5) and HAS-BLED (median = 3) than those on OAT (median age 82.9, CHA_2_DS_2_-VASc = 4, HAS-BLED = 2). Comparison between patients according to admission preventive therapy (OAT vs. no preventive therapy) is shown in Table 1.

### 4.3. Secondary Prevention Therapy at Discharge and Follow-Up

**Figure 1:**
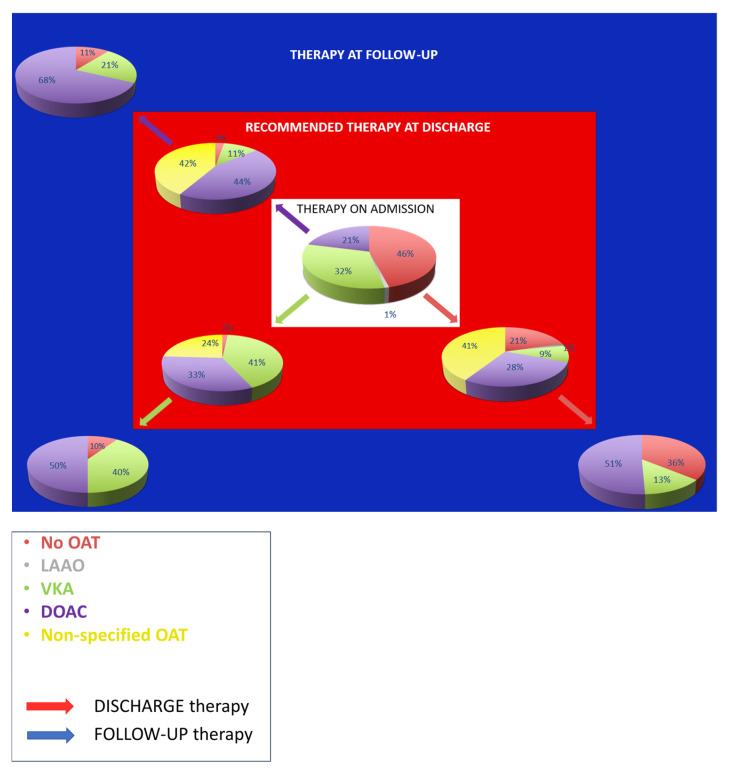
Secondary prevention strategies according to the prevention therapy on admission. Abbreviations: OAT, Oral Anticoagulant Therapy; LAAO, Left Atrial Appendage Occlusion; VKA, Vitamin K Antagonist; DOAC, Direct Oral Anticoagulant.

Figure 1 shows the secondary prevention strategies at discharge and at follow-up according to the preventive therapy on admission.

In detail among patients without preventive therapy at admission (46%, 103/224), 78.4% (69/88) were prescribed OAT at discharge, but 36% (27/75) were not taking OAT at follow-up. Of those on DOAC at admission (21%, 47/224): 44.4% (16/36) were advised to continue DOAC, 11.1% (4/36) to shift to VKA and 41.7% (15/36) to OAT without further indication of the type; at follow-up 10.7% (3/28) were not taking OAT. Among patients on VKA at admission (32%, 72/224): 41.4% (24/58) were advised to continue VKA, 32.8% (19/58) to switch to DOAC, 24.1% (14/58) to non-specified OAT; at follow-up, 9.5% (4/42) were off OAT.

### 4.4. In-Hospital Outcomes

During the hospitalization 20 patients (8.8%) died; their pre-admission therapy was VKA in 7, DOAC in 8, no prevention in 5. The median NIHSS in these patients was 19.6, and pre-event mRS was >2 in 11 patients.

### 4.5. Follow-Up Outcomes

Among the remaining 206 patients, 24 were lost at follow-up. Median follow-up duration was 208 (range 85–443) days.

Fifty-seven patients died during follow-up, with causes including IS in 25 (43.9%), haemorrhagic stroke in 2 (3.5%), other causes in 6 (10.5%), and unknown in 24 (42.1%). Table 2 reports comparison of clinical characteristics by survival status at follow-up. In the multivariable model, age (OR 1.22, 95% CI 1.10–1.36, *p* < 0.001), admission NIHSS (OR 1.13, 95% CI 1.06–1.21, *p* < 0.001), and diabetes (OR 5.72, 95% CI 1.39–23.57, *p* = 0.016) remained independently associated with an increased risk of death during follow-up. Eighteen patients experienced a new cerebrovascular event (13 ischaemic stroke, 2 TIA, 1 haemorrhagic stroke, 1 unknown): 7 patients were on DOAC (3 ischaemic stroke, 2 TIA, 1 haemorrhagic stroke, 1 unknown), 2 were on VKA, 7 did not assume OAT, and 2 on unknown therapy.

**Figure 2:**
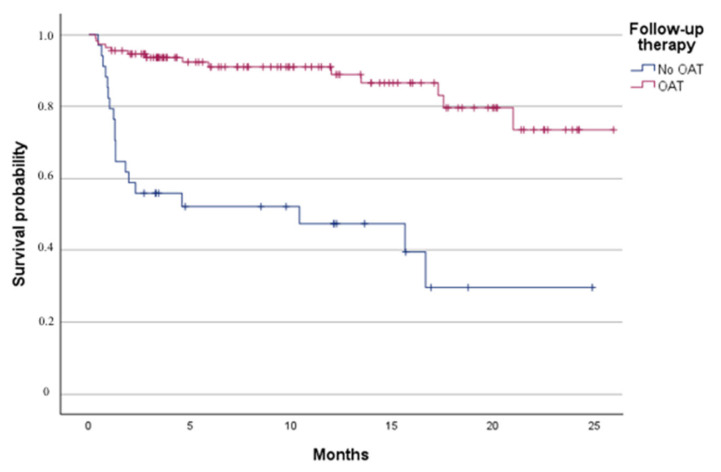
Survival probability according to follow-up secondary prevention therapy (Kaplan Meier analysis). Abbreviations: OAT, Oral Anticoagulant Therapy.

Among 146 patients with known therapy at follow-up survival was significantly higher in those on OAT (*p* < 0.001) (Figure 2). This association remained significant after adjustment for age and NIHSS at admission (OR 2.95, 95% CI 1.20–7.25, *p* = 0.018). In a sensitivity analysis restricted to 98 patients with available pre-event mRS, the association was no longer statistically significant (OR 0.50, 95% CI 0.13–1.16, *p* = 0.224).

### 4.6. Adequacy of OAT and Resistant Stroke

According to our criteria OAT resulted adequate in 62 patients (52.1%), non-adequate in 45 (37.8%), and uncertain in 12 (10.1%).

Table 3 shows the comparison between patients on adequate vs. non adequate OAT.

**Figure 3:**
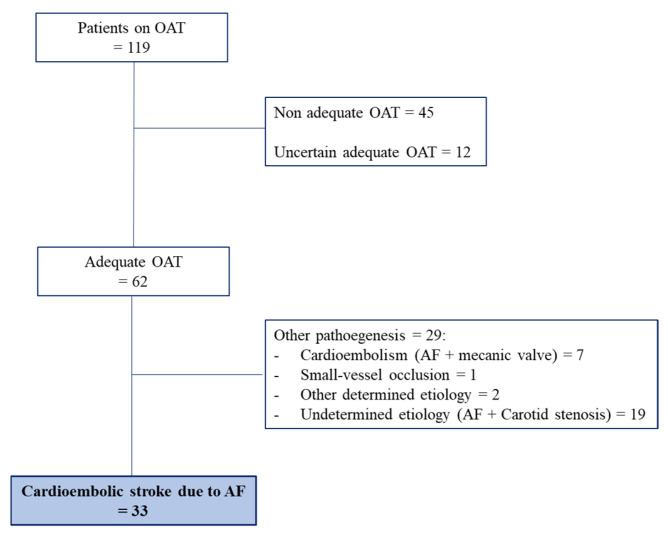
Identification of patients with ischaemic cardioembolic stroke due to AF despite adequate OAT (Resistant Stroke). Abbreviations: OAT, Oral Anticoagulant Therapy; AF, Atrial Fibrillation.

Thirty-three (53%) out of the 62 patients on adequate OAT were found to have a RS (Figure 3). One of them died during hospitalization.

Secondary stroke prevention therapy at discharge for the remaining 32 patients is shown in Table 4.

## 5. Discussion

Our study provides a snapshot of the therapeutic management of AF in both primary and secondary prevention of IS. Nearly half of the patients were not receiving anticoagulants at admission, yet among these 78.4% were prescribed OAT at discharge, suggesting that in most cases a strong contraindication was absent (Figure 1).

However, the improvement observed at discharge was not sustained over time: while only 11% of patients were not advised to OAT, this proportion rose to 23.3% at follow-up. These findings, in line with previous reports, reflect the complexity of OAT management [10] and the persistent concerns among clinicians about bleeding risk—particularly in elderly and frail patients, for whom the risks of intracranial haemorrhage, falls, and polypharmacy are heightened. Such concerns often lead to under prescription or intentional underdosing of OAT, without a clear clinical indication. While dose reduction is appropriate in specific scenarios—like impaired renal function or low body weight—recent studies have shown that inappropriate DOAC underdosing is associated with increased thromboembolic risk without a proportional reduction in bleeding events [11]. A comprehensive, multidisciplinary assessment is critical when prescribing OAT in the ederly. In selected patients, such as those with limited life expectancy or severe frailty, withholding OAT may be appropriate, as highlighted by EHRA recommendations [9]. In this context, greater awareness of LAAO as a validated alternative for selected subgroups may help address underuse of OAT [12].

Among patients on anticoagulants, almost 40% had non-adequate adherence, and a prior history of ischaemic events did not significantly increase prescription or adherence rates. Approximately three-quarters of patients on non-adequate OAT experienced AF related cardioembolic stroke, events that could potentially have been prevented through evidence-based prescribing and closer compliance monitoring. This underscores the need for systematic follow-up, dedicated anticoagulant services, and stronger involvement of family and social support networks to promote adherence and continuity of care.

In our cohort, mortality at follow-up was independently associated with older age, greater stroke severity at admission, and diabetes [13], confirming the substantial mortality and disability burden of AF-related IS.

OAT use was linked to lower mortality at follow-up, however, this finding should be interpreted with caution due to the limited sample size and potential selection bias—healthier patients are more likely to receive OAT, whereas frailer individuals often have it withheld. In our sensitivity analysis, inclusion of baseline disability as a covariate attenuated the association between OAT and survival, suggesting that functional status before stroke may be a key confounder. The reduced sample size in this adjusted model likely further contributed to the loss of statistical significance.

Regarding cardiovascular risk factors, no significant differences emerged between adequate and non-adequate OAT, except for dyslipidemia. Recent study suggests that hyperlipidaemia may play a role in the occurrence of ischemic stroke despite DOAC adequately conducted [14], warranting further investigation.

The variability of the recommended therapy at discharge among patients already on OAT at admission reflects the challenges and empirical nature of therapeutic decisions in this setting. Current guidelines do not provide clear diagnostic or therapeutic algorithms for such patients [12,15], and existing studies suggest that switching OAT does not necessarily reduce recurrence risk [10,16]. However, these studies did not stratify patients by the underlying cause of stroke, suggesting that switching anticoagulants may not be necessary when the cerebral event is due to a non-AF-related mechanism. In fact, not all strokes in AF patients should be considered OAT failures: in our cohort, approximately 30% of patients had an additional stroke mechanism, reinforcing the importance of a thorough etiological work-up to identify alternative causes, which may warrant different or combined prevention strategies.

Our study aligns with recent data, showing a high rate of RS [17], underscoring the potential failure of OAT in stroke prevention, as previously cautioned in the existing literature [18,19,20]. Although we refer to these events as RS, the concept closely relates to what is often termed “anticoagulation failure” in the international literature. However, a universally accepted definition is lacking, and current studies adopt heterogeneous criteria to classify such cases—ranging from any ischemic event occurring during OAT to only those with objectively verified therapeutic anticoagulation at the time of stroke. By adopting an explicit definition of this phenomenon, our study aims to contribute to a clearer operational framework for both research and clinical care.

An underexplored aspect in the context of RS is the potential contribution of Left Atrial Appendage (LAA) morphology. Up to 90% of thrombi in AF patients originate from the LAA, and four morphologies—chicken-wing, windsock, cactus, and cauliflower—have been described, each with different thrombogenic potentials. [21]. Features such as large volume, low flow velocity, many trabeculation, multiple lobes and large orifice diameter may, also, contribute to further increase thrombotic risk [22,23]. A “malignant” morphology may help explain embolic events despite well-conducted anticoagulation. Incorporating dedicated LAA imaging (e.g., transesophageal echocardiography, cardiac CT) into RS workup could improve recurrence risk stratification and support consideration of interventions, such as LAAO add-on to OAT.

Strokes related to AF represent not only a significant clinical challenge but also a major economic burden for healthcare systems, often resulting in prolonged hospitalization, extensive rehabilitation, and long-term disability care. Improving anticoagulation management and effectively preventing strokes could therefore reduce both clinical complications and the associated financial impact [24].

Our study has limitations and strengths that need to be addressed. The single centre design and small sample size limit the generalizability of the findings. As a hub centre, many patients are transferred from spoke hospitals for endovascular treatment and return to the referring centre within 48 h, resulting in incomplete discharge data for some cases. The retrospective design precluded a detailed evaluation of DOAC use in certain patients and led to the exclusion of individuals with uncertain compliance; we exclude this group to ensure a clearly defined study population; nevertheless, this approach may have introduced selection bias. For VKA-treated patients, in most of them only emergency department INR value was available to assess adoption of the therapy. Additional follow-constraints included missing data, variable follow-up intervals, and an almost 10% loss to follow-up. The observed survival advantage among patients receiving OAT may be affected by selection bias and residual confounding from baseline functional status, which was only available for a subset of patients. Considering the small sample size in the RS subgroup, our analysis was restricted to a descriptive report of therapeutic management at discharge and was not designed or powered to evaluate clinical outcomes. Despite these limitations, our study provides valuable real-world insights into the therapeutic management of AF for ischaemic stroke prevention.

## 6. Conclusions

In conclusion our study highlights two key points:

(1) the suboptimal adoption of international recommendations regarding OAT and the challenges clinicians faces in balancing its risks and benefits, underscoring the need for evaluation in specific centres. This is particularly evident in elderly patients, where both under prescription and inappropriate underdosing remain common, often driven by an overestimation of bleeding risk. A multidisciplinary approach involving neurologists, cardiologists, geriatricians, and primary care physicians may further enhance outcomes in this population.
(2) RS is not an uncommon condition with an as-yet-unknown mechanism, hence leading to an empirical therapeutic approach. Further research should focus on systematic etiological workups in RS, including LAA morphology. Such approaches could help identify RS patients at higher residual risk and guide more tailored preventive strategies.

## Figures and Tables

**Table 1:** Patients’ characteristics in the whole group and according to admission therapy.

		OAT		
	*n* = 226	Yes (*n* = 119)	No (*n* = 103)	*p*
Age, years (median, IQR)	84.04 (77.9–88.6)	82.9 (77.3–87.6)	85.3 (78.1–90.0)	0.077
Sex (F)	128/226 (61%)	72/119 (60.5%)	64/103 (62.1%)	0.803
Hypertension	172/224 (76.7%)	31/118 (26.3%)	20/103 (19.4%)	0.228
Diabetes	46/223 (20,6%)	26/117 (22.2%)	20/103 (19.4%)	0.610
Dyslipidaemia	66/152 (43.4%)	33/80 (41.3%)	33/70 (47.1%)	0.468
Actual smoking habit	10/158 (6.3%)	3/86 (3.5%)	7/71 (9.9%)	0.104
Previous smoking habit	62/158 (39.2%)	40/86 (46.5%)	32/71 (45.1%)	0.875
Previous ischaemic stroke	41/220 (18.6%)	16/62 (25.8%)	7/44 (15.9%)	0.223
Previous haemorrhagic stroke	14/221 (6.3%)	3/62 (4.8%)	1/44 (2.3%)	0.495
Heart failure	63/219 (28.8%)	21/62 (33.9%)	16/44 (36.4%)	0.791
CHA_2_DS_2_-VASc (median, IQR)	4.5 (3–6)	4 (3–6)	5 (3–6)	0.774
HAS-BLED (median, IQR)	3 (2–3)	3 (2–3)	2 (2–3)	**0.007**
mRS pre-stroke (median, IQR)	2 (0–3)	1 (0–3)	2 (0–3)	0.794
Ischaemic cerebrovascular event TIA Ischaemic Stroke	8/226 (3.5%) 218/226 (96.5%)	3/229 (2.5%) 116/119 (97.5%)	5/103 (4.9%) 98/103 (97.5%)	0.352
TOAST Cardioembolic Small-artery occlusion Other determined cause Indeterminate (*AF* + *atherothrombosis*)	159/226 5/226 (2.2%) 2/226 (0.8%) 60/226 (26.5%)	74/119 (62.2%) 3/119 (2.5%) 9/119 (7.6%) 33/119 (27.7%)	74/103 (71.8%) 2/103 (1.9%) 0/103 (0.0%) 27/103 (26.2%)	**0.034**
NIHSS on admission (median, IQR)	15 (6–21)	15.5 (6–21)	13.5 (5–21)	0.271
Systemic thrombolysis	49/216 (22.7%)	1/59 (1.7%)	12/43 (27.9%)	**<0.001**
Mechanical thrombectomy	78/216 (36.1%)	27/59 (45.8%)	13/43 (30.2%)	0.113

Abbreviations: OAT, Oral Anticoagulant Therapy; IQR, Interquartile Range; F, Female; TIA, Transient Ischaemic Attack; AF, Atrial Fibrillation; NIHSS, National Institutes of Health Stroke Scale.

**Table 2:** Comparison of clinical characteristics by survival status at follow-up.

	Death During Follow-Up		Univariate		Multivariate	
	Yes (*n* = 57)	No (*n* = 125)	OR (95% CI)	*p*	OR (95% CI)	*p*
Age, years (median, IQR)	88.1 (82.3–90.8)	86.2 (74.4–90.5)	**1.13 (1.07–1.19)**	**<0.001**	**1.22 (1.10–1.36)**	**<0.001**
Sex (F)	40.4% (23/57)	42.4% (53/125)	1.09 (0.58–2.06)	0.795		
NIHSS on admission	18 (10–22)	9 (3.5–17.5)	**1.09 (1.04–1.14)**	**<0.001**	**1.13 (1.06–1.21)**	**<0.001**
Hypertension	84.2% (48/57)	73.6% (92/125)	1.91 (0.85–4.32)	0.119		
Diabetes	29.8% (17/57)	16.8% (21/125)	**2.11 (1.01–4.39)**	**0.048**	**5.72 (1.39–23.57)**	**0.016**
Dyslipidaemia	31.6% (12/38)	47.8% (44/92)	0.50 (0.23–1.12)	0.092		
mRS pre-stroke	2 (0–4)	0 (0–3)	**1.33 (1.04–1.68)**	**0.022**	0.96 (0.67–1.39)	0.840
CHA_2_-DS_2_-VASC	5 (4–6)	4 (3–6)	**1.40 (1.31–1.74)**	**0.002**	1.03 (0.65–1.64)	0.891
HAS-BLED	3 (2–3)	2 (2–3)	**1.60 (1.12–2.29)**	**0.010**	1.88 (0.88–3.96)	0.103

For continuous variables, the OR refers to the increase per unit; for age = per 1-year increase; for NIHSS = per 1-point increase. Abbreviations: IQR, Interquartile Range; F, Female; NIHSS, National Institutes of Health Stroke Score.

**Table 3:** Comparison between patients on adequate and non-adequate OAT.

	Adequate OAT		
	Yes (*n* = 62)	No (*n* = 45)	*p*
Age, years (median, IQR)	82.2 (73.9–87.0)	84.1 (79.2–88.4)	0.118
Sex (F)	23/62 (37.1%)	21/45 (21%)	0.321
Hypertension	44/62 (72.6%)	32/44 (72.7%)	0.987
Diabetes	14/62 (22.6%)	8/44 (18.2%)	0.582
Dyslipidaemia	23/43 (53.5%)	8/33 (24.2%)	**0.010**
Actual smoking habit	2/42 (4.8%)	1/36 (2.8%)	0.650
Previous smoking habit	23/42 (54.8%)	16/36 (44.4%)	0.364
Obesity	4/27 (14.8%)	4/13 (30.8%)	0.237
Previous ischaemic stroke	16/62 (25.8%)	7/44 (15.9%)	0.223
Previous haemorrhagic stroke	3/62 (4.8%)	1/44 (2.3%)	0.495
Heart failure	21/62 (33.9%)	16/44 (36.4%)	0.791
CHA_2_DS_2_-VASc (median, IQR)	5 (3–6)	4 (3–6)	0.437
HAS-BLED (median, IQR)	3 (2–3)	3 (2–3)	0.951
Ischaemic cerebrovascular event TIA Ischaemic Stroke	1/62 (1.6%) 61/62 (98.4%)	2/45 (4.4%) 43/45 (95.6%)	0.381
TOAST Cardioembolic AF AF + mechanic prothesis Small-artery occlusion Other determined cause Indeterminate (*AF* + *atherothrombosis*)	40/62 (64.5%) 33/40 (82.5%) 7/40 (17.5%) 1/62 (1.6%) 2/62 (3.2%) 19/62 (30.6%)	33/45 (73.9%) 31/33(93.9%) 2/33 (6.1%) 1/45 (2.2%) 0/45 (0.0%) 11/45 (24.4%)	0.540
NIHSS on admission (median, IQR)	11 (5–20)	16 (6–21)	0.357
Systemic thrombolysis	1/59 (1.7%)	12/43 (27.9%)	<0.001
Mechanical thrombectomy	27/59 (45.8%)	13/43 (30.2%)	0.113
Transthoracic echocardiography	50/62 (80.6%)	40/45 (88.9%)	0.250
Atrial enlargement	40/45 (88.9%)	35/40 (87.5%)	0.843
Normal ejection fraction	31/46 (67.4%)	26/36 (72.2%)	0.637
Mitral valvulopathy Stenosis Insufficiency Steno-insufficiency	0/50 (0.0%) 34/50 (68.0%) 2/50 (4.0%)	1/39 (2.6%) 27/39 (69.2%) 2/39 (5.1%)	0.629
Aortic Valvulopathy Stenosis Insufficiency Steno-insufficiency	4/50 (8.0%) 15/50 (30.0%) 2/50 (4.0%)	2/39 (5.1%) 19/39 (46.2%) 5/39 (10.3%)	0.189
Mitral prosthetic heart valves Mechanic Biologic	5/62 (8.1%) 7/62 (11.3%)	2/43 (4.5%) 0/43 (0.0%)	0.052
Aortic prosthetic heart valves Mechanic Biologic	3/62 (4.8%) 10/62 (16.1%)	2/43 (4.7%) 1/43 (2.3%)	0.074

Abbreviations: OAT, Oral Anticoagulant Therapy; IQR, Interquartile Range; F, Female; TIA, Transient Ischaemic Attack; AF, Atrial Fibrillation; NIHSS, National Institutes of Health Stroke Scale.

**Table 4:** Secondary prevention therapy at discharge in patients with RS.

		Recommended Therapy at Discharge		
		VKA	DOAC	Non-Specified OAT
Therapy on Admission	VKA (*n* = 20)	45% (9/20)	45% (9/20)	10% (2/20)
	DOAC (*n* = 12)	16.7% (2/12)	66.6% * (8/12)	16.7% (2/12)

* Five patients shifted from one DOAC to another, in particular 2 from factor IIa inhibitor to factor Xa inhibitor, 3 from factor Xa inhibitor to factor IIa inhibitor. Three patients maintained with the same DOAC. Abbreviations: VKA, Vitamin K Antagonist; DOAC, Direct Oral Anticoagulant; OAT, Oral Anticoagulant Therapy.

## Data Availability

The original contributions presented in this study are included in the article. Further inquiries can be directed to the corresponding author(s).

## Abbreviations

The following abbreviations are used in this manuscript:

AF	Atrial Fibrillation
DOAC	Direct Oral Anticoagulant
IS	Ischemic Stroke
LAA	Left Atrial Appendage
LAAO	Left Atrial Appendage Occlusion
OAT	Oral Anticoagulant Therapy
RS	Resistant Stroke
TIA	Transient Ischemic Attack
VKA	Vitamin K Antagonist
